# Metal Oxide/Nitrogen-Doped Carbon Nanosheet Heteronanostructures as Highly Efficient Electromagnetic Wave Absorbing Materials

**DOI:** 10.3390/molecules26247537

**Published:** 2021-12-13

**Authors:** Yilin Huang, Weidong Xue, Xingwang Hou, Rui Zhao

**Affiliations:** School of Materials and Energy, University of Electronic Science and Technology of China, Chengdu 610054, China; hyl7605228132021@163.com (Y.H.); xuewd2008@163.com (W.X.); xingwanghou@outlook.com (X.H.)

**Keywords:** metal phthalocyanine, carbon nanosheet, solid-state pyrolysis, electromagnetic wave absorption

## Abstract

In this paper, we will discuss the excellent broadband microwave absorption behaviors of Cu/CuO/carbon nanosheet composites: traces of copper and oxide embedded in a carbon nano-sheet not only cut down the high permittivity of adsorbs but also induce more interfacial polarization centers. The results showed that at a cracking temperature of 900 °C, the fabricated material has a unique ripple-like structure, which promotes the hierarchical interfacial polarization. The prepared material has a maximum absorption bandwidth of 4.48 GHz at an exceedingly thin thickness of 1.7 mm and a maximum reflection loss of −25.3 dB at a thickness of 2 mm. It is a relatively ideal material for electromagnetic wave absorption.

## 1. Introduction

Bulk synthesis of low-dimensional carbon materials from a simple, controllable, and low-cost method is still a critical target in scientific and industrial research. Solid-state pyrolysis of organometallic compound precursors has recently emerged as an alternative method for preparing carbon nanostructures, such as carbon nanotubes (CNTs) and amorphous carbon [1,2,3,4,5]. Being superior to the traditional CVD method, which is particularly attractive for the synthesis of aligned CNTs in the gas phase, controllable solid-state pyrolysis of organometallic precursors does not need a supply of hydrogen to maintain the activity of the catalysts during the high-temperature process (800–1300 °C). In such a process, the organometallic complexes serve as both the catalyst precursors and the carbon source, and the pyrolysis is carried out not in the gas phase but in the solid state at “low” temperatures.

Today, the widespread application of wireless devices has become an irresistible trend. Despite tremendous efforts, fabrication of lightweight conductive fabrics for high-performance X-band (ranging from 8 to 12 GHz) electromagnetic-interference (EMI) shielding remains a daunting technical challenge [6,7,8,9]. Carbon-based materials such as carbon nanotube (CNT), graphene, graphene oxide (GO), and reduced graphene oxide (rGO) have the advantages of corrosion resistance, lightweight, excellent flexibility, and easy fabrication, so they have been widely proposed as ideal EMI shielding materials [10,11,12,13,14,15,16,17]. However, sole carbon materials suffer from improper electrical conductivity and a limited electromagnetic wave attenuation mechanism. Incorporation of other lossy materials has been widely studied as the imperative solution to improve their microwave loss performance and expand their absorber bandwidth.

Phthalocyanines (Pcs) are planar aromatic macrocycles consisting of four isoindole units presenting an 18-electron aromatic cloud delocalized over an arrangement of alternated carbon and nitrogen atoms. They have a wide range of applications in many areas [18,19,20,21,22,23,24,25]. Due to the extremely high thermal decomposition temperature, high char yields, and greater than 19 wt% of nitrogen atoms, in situ synthesis of metal oxide/nitrogen doping graphene in bulk by the pyrolysis of copper phthalocyanine has been reported in our previous research work [26]. In this manuscript, we will focus on discussing the excellent broadband microwave absorption behaviors of Cu/CuO/carbon nanosheet composites: traces of copper and oxide embedded in the carbon nano-sheet not only reduced the high permittivity properties of adsorbs but also induced more interfacial polarization centers without sacrificing the corrosion resistance of the carbon materials. The prepared material has a maximum absorption bandwidth of 4.48 GHz at an exceedingly thin thickness of 1.7 mm and a maximum reflection loss of −25.3 dB at a thickness of 2 mm. It is a relatively ideal material for electromagnetic wave absorption.

## 2. Results and Discussion

**Figure 1:**
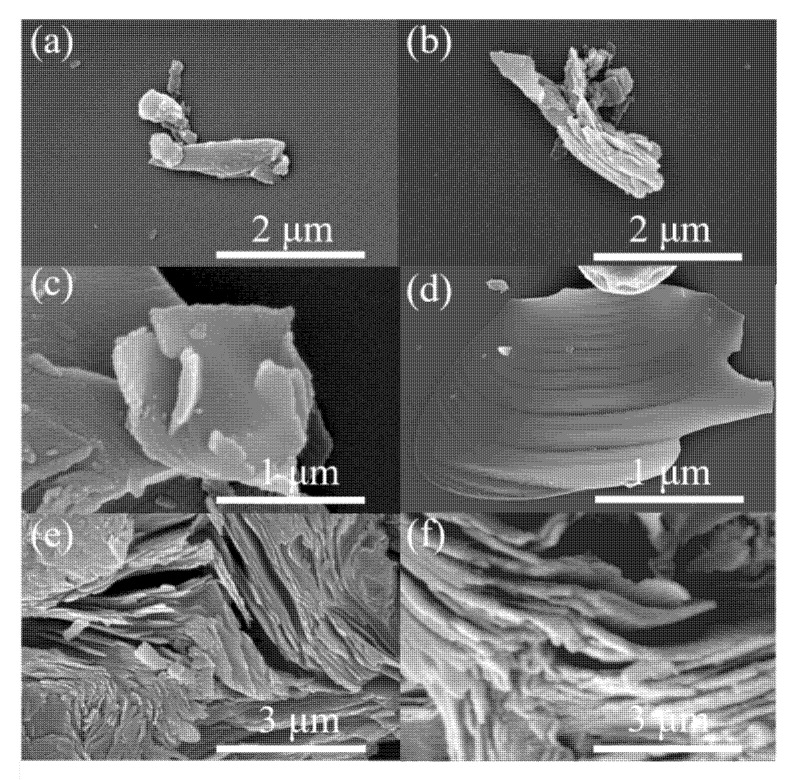
SEM images of (**a**) CuPc-600 (**b**) CuPc-700 (**c**) CuPc-800 (**d**,**e**) CuPc-900 (**f**) CuPc-900/HCl.

Figure 1 shows the SEM images of copper phthalocyanine carbon nanosheets. When the pyrolysis temperature is 600 °C, CuPc is cleaved into many small, thick block structures (Figure 1a). When the pyrolysis temperature is 700 °C, the block structure shows some signs of cleavage, and it can be clearly observed that the block structure has changed into a multi-layered stacked structure (Figure 1b). The structure changed significantly when the cracking temperature was increased to 800 °C. At this time, a square sheet structure with a length of about 1 μm has been produced; however, from the side of the curled layer, it can be observed that the thickness of the layer is still at a thicker level (Figure 1c). The structure of the copper phthalocyanine carbon nanosheets was further improved when the cleavage temperature was increased to 900 °C. Compared with CuPc-800, CuPc-900 has thinner lamellae and, also, has a corrugated structure (Figure 1d). Figure 1e is a side view of an incomplete cleavage of CuPc-900. It can be clearly seen that the materials present a lamellar structure and are stacked in multiple layers. The combination of this unique corrugated structure and the stacked multilayer carbon nanosheet structure increases the specific surface area and plays a substantial role in the reflection of electromagnetic waves. Furthermore, the structure with gaps between the layers also may help the multiple reflections of electromagnetic waves and enhance the electromagnetic wave loss capacity of the material, so as to advance the wave absorption capacity of the material. Figure 1f shows the SEM image of CuPc-900/HCl. After the immersion in hydrochloric acid, it can be observed that the laminar stacking structure is not destroyed.

**Figure 2:**
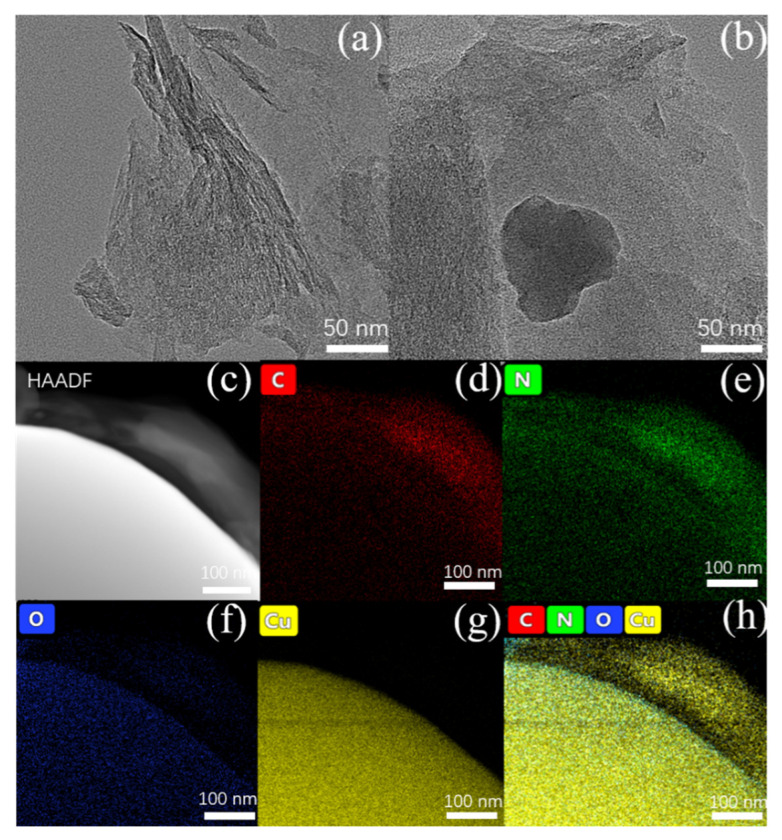
(**a**,**b**) TEM images of copper phthalocyanine after 900 °C temperature treatment. (**c–h**) HAADF image and the corresponding EDS Cu, O, C, and N mapping of copper phthalocyanine after 900 °C temperature treatment.

**Figure 3:**
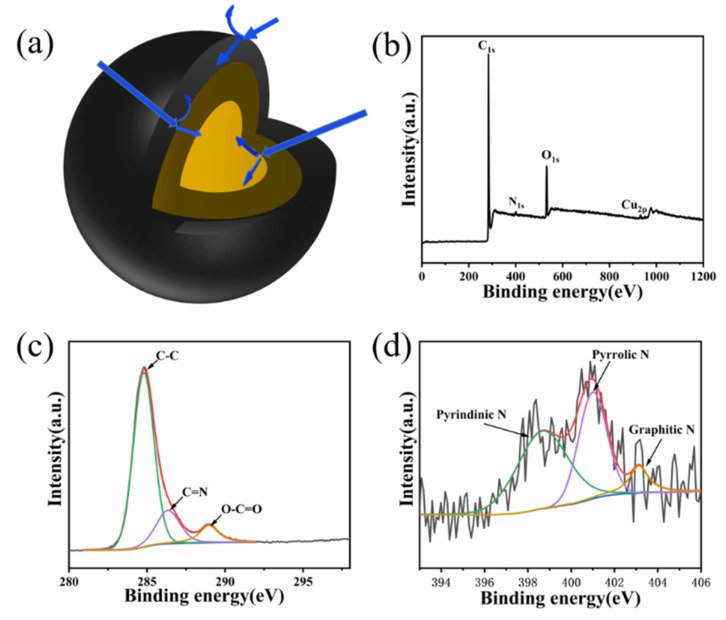
Schematic diagram of material structure (**a**) and XPS spectra of CuPc-900 of (**b**) survey scan: (**c**) C 1s; (**d**) N 1s.

Figure 2 shows the TEM pattern of CuPc-900. The image shows that CuPc-900 is a thin lamellar structure with a size of about a few hundred nanometers; this also coincides with the previous SEM images. In addition to this, there are copper particles interspersed between some lamellar layers. Figure 2c–h demonstrates the TEM and the corresponding EDS Cu, C, O, and N mappings. It can be seen that C and N are uniformly distributed in the graphene lamellae region, while the regions of O and Cu are basically overlapping, indicating that the O element is mainly combined with Cu to form copper oxides. It can be inferred that CuPc-900 may have three interfaces, carbon nanosheets, copper oxide, and copper, which can reflect electromagnetic waves. As shown in the schematic diagram of the model in Figure 3a, the outermost layer is the graphene sheet layer in which the copper particles are wrapped, and the surface of the copper particles has a layer of copper oxide, forming an overall three-layer structure. When the incident electromagnetic wave comes in, it will first be reflected and transmitted in the outermost graphene sheet layer, and the electromagnetic wave transmitted into the interior of the material will be reflected and transmitted on the surface of the copper oxide. The electromagnetic wave transmitted into the copper particles in this step will be reflected and projected again, and there are interface reflection losses between the three interfaces, which can change the loss mechanism of the material for electromagnetic waves, and thus greatly improve the absorption performance of electromagnetic waves.

To further investigate the elemental composition and valence of CuPc, XPS was used to study CuPc. As shown in Figure 3b, CuPc-900 consists of C, N, O, and Cu, and their contents are 85%, 2.47%, 12.17%, and 0.36%, respectively. Compared to CuPc as a precursor, the C content is higher and the N content is lower, which should be caused by the denitrification during the cracking process. While CuPc does not contain elemental oxygen, the presence of elemental oxygen in the final sample should be caused by the adsorbed oxygen in the material and the unexhausted oxygen in the tube furnace. The abnormally low elemental copper content is due to the enhanced tendency of some metal atoms to agglomerate into nanoparticles at elevated temperatures, resulting in a reduced surface exposure area; some are encapsulated by carbon nanosheets, resulting in a corresponding decrease in the characterized content. In Figure 3c, the three peaks in the C 1s spectra correspond to C-C (284.8 eV), C=N (286.4 eV), and C=O (289.0 eV). The peak splitting of element N is shown in Figure 3d where 398.5 eV, 401.0 eV, and 403.1 eV correspond to pyridine type nitrogen, pyrrole type nitrogen, and graphite type nitrogen, respectively.

**Figure 4:**
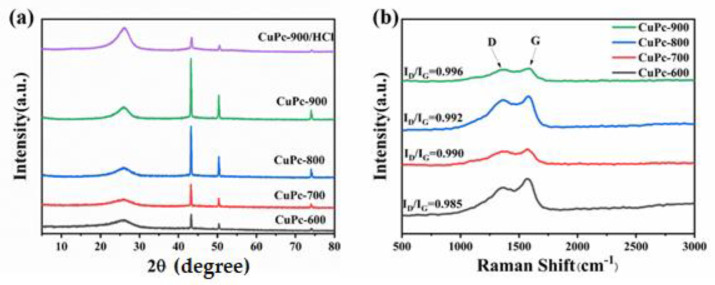
XRD patterns of CuPc-600, CuPc-700, CuPc-800, CuPc-900, and CuPc-900/HCl (**a**) and Raman patterns of CuPc-600, CuPc-700, CuPc-800, and CuPc-900 (**b**).

As shown in Figure 4a, the XRD analysis of CuPc-600, CuPc-700, CuPc-800, and CuPc-900 shows that all samples have a broad characteristic diffraction peak of the graphite (002) crystalline surface around 26°. It indicates that the sample appears graphitized and the peaks become sharper as the cleavage temperature increases. In addition, the characteristic diffraction peaks of Cu (Jade:PDF#04-0836) are evident at 43.21°, 50.31°, and 74.08°, and the higher the cracking temperature, the sharper the corresponding diffraction peaks, just like the graphite peak at 26°. The above XRD results indicate that CuPc forms graphite-type layered carbon materials when it is cleaved at 600 °C and above and that the metallic Cu in the center is reduced to Cu monomers. This phenomenon becomes more pronounced as the cleavage temperature increases. The characteristic peaks of Cu of CuPc-900/HCl are obviously weaker than those of CuPc-900, which indicates that the copper and copper derivatives have been cleaned by hydrochloric acid. Combined with the previous SEM images, it can be shown that if there is a change in the absorption performance, it should be caused by the destruction of the triple interface of Cu/CuO/carbon. Figure 4b shows the Raman pattern of the samples. From the Raman pattern, we can observe the change in the degree of graphitization of the material, and it can be found that all the four samples have two characteristic peaks, the D peak and G peak, which correspond to defective carbon (sp^3^) and graphitic carbon (sp^2^), respectively. The values of I_D_/I_G_ for CuPc-600, CuPc-700, CuPc-800, and CuPc-900 are 0.985, 0.990, 0.992, and 0.996, respectively. With the increase in treatment temperature, the disorder of carbon increases and more defects are formed in the samples, which may promote the formation of more dipoles and enhance the polarization loss of microwaves.

**Figure 5:**
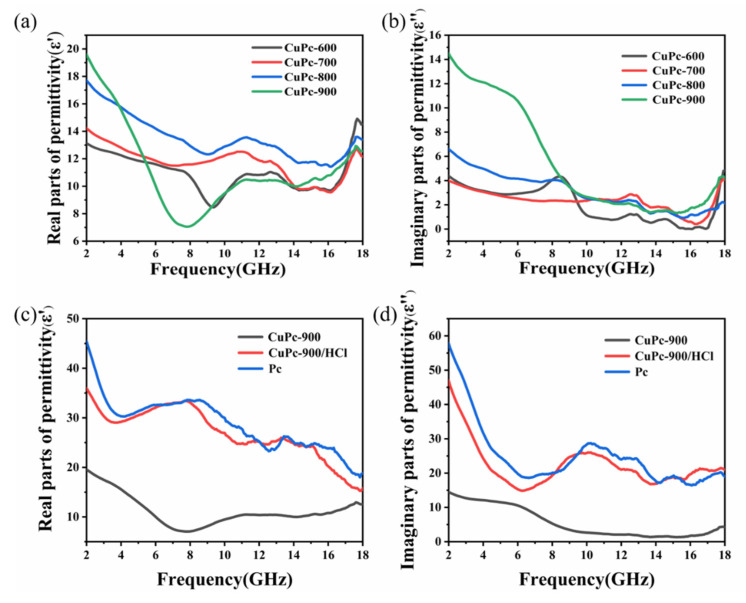
Comparison graph of dielectric constants of copper phthalocyanine carbon nanosheets. (**a**,**c**) Real parts of permittivity and (**b**,**d**) imaginary parts of permittivity.

The complex permittivity (*ε*_r_ = *ε*′ − j*ε*″) and the complex permeability (*μ*_r_ = *μ*′ − j*μ*″) of a material are important measures of its wave absorption properties. Among them, *ε*′ and *μ*′ represent the real part of the complex permittivity and complex permeability, reflecting the ability of the material to store electromagnetic waves; *ε*″ and *μ*″ represent the imaginary parts of the complex permittivity and magnetic permeability, which reflect the ability of the material to lose electromagnetic waves. For the carbon material prepared in this paper, it has almost no magnetic loss capability, so only the dielectric loss capability of the material is discussed. The electromagnetic parameters of the paraffin mixtures of copper phthalocyanine carbon nanosheets with a mass fraction of 50% were measured using a vector network analyzer. The real and imaginary parts of CuPc-600, CuPc-700, CuPc-800, and CuPc-900 are shown in Figure 5a,b, respectively. It can be seen that as the processing temperature increases, the starting *ε*′ and *ε*″ values are also increasing with increasing treatment temperature, where the three samples CuPc-600, CuPc-700, and CuPc-800 show approximately the same trend. The pattern for CuPc-900 appears somewhat different, in the range of 2–8 GHz *ε*′ drops from 19.6 to 7, much lower than the other three samples. *ε*″ starts at 14, which is about two to three times higher than the other samples, and in the 2–6 GHz range, *ε*″ decreases more slowly from 14 to 11. In the 6–8 GHz range, it drops steeply from 11 to about 4, reaching about the same level as the other three samples. Figure 5c,d show the comparison of the real and imaginary parts of the dielectric constants of CuPc-900, CuPc-900/HCl, and Pc. It can be seen that compared to CuPc-900/HCl and Pc, the real and imaginary parts of the dielectric of CuPc-900 are very low, indicating that the special structure of Cu/CuO/carbon nanosheet composites plays a role in reducing the dielectric of the material, and thus regulating its impedance matching. The overall trend of CuPc-900/HCl is similar to that of Pc, but its starting value is a little lower than that of Pc. The previous XRD characterization results show that the CuPc-900 washed with HCl still contains a small amount of copper that has not been washed off, which further confirms the dielectric-lowering effect of copper. The possible mechanism is that the carbon nanosheet can be regarded as a whole conductive network, while copper and copper oxide are equivalent to a small resistance, and the current through the equivalent resistance will change its current loss mechanism, thus playing a role in improving the impedance matching.

**Figure 6:**
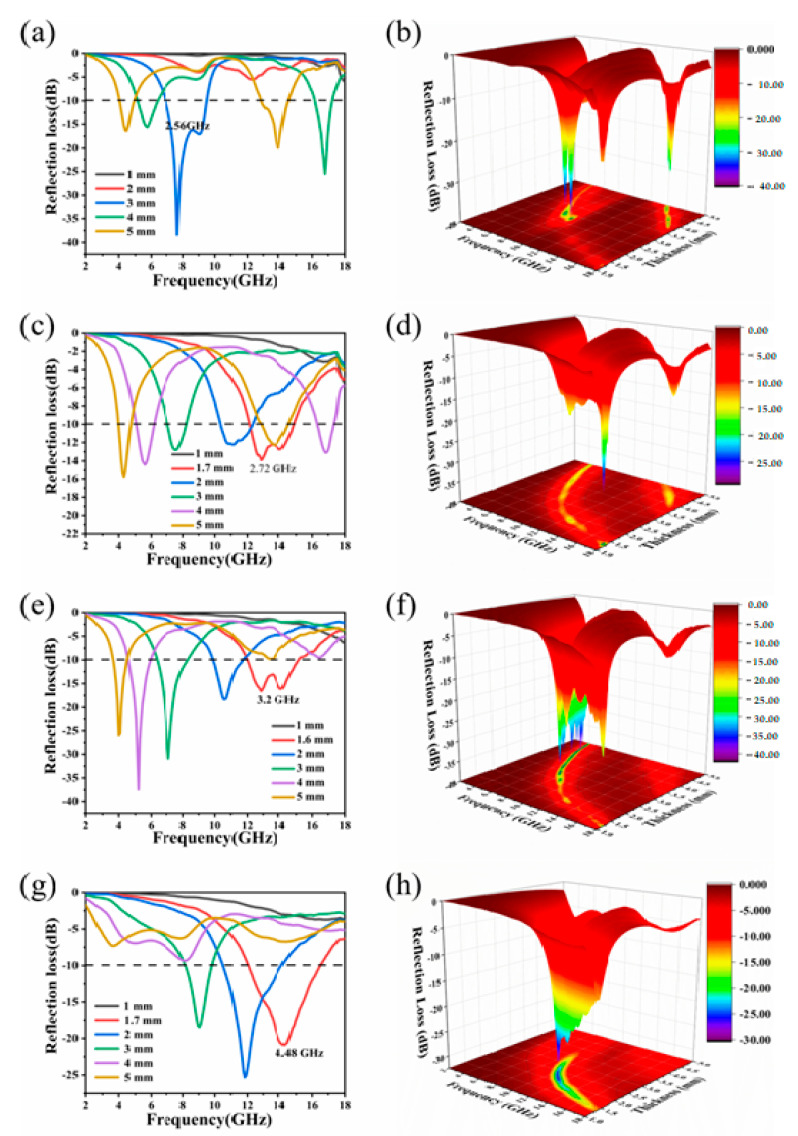
Reflection loss of (**a**,**b**) CuPc-600, (**c**,**d**) CuPc-700, (**e**,**f**) CuPc-800, and (**g**,**h**) CuPc-900.

**Figure 7:**
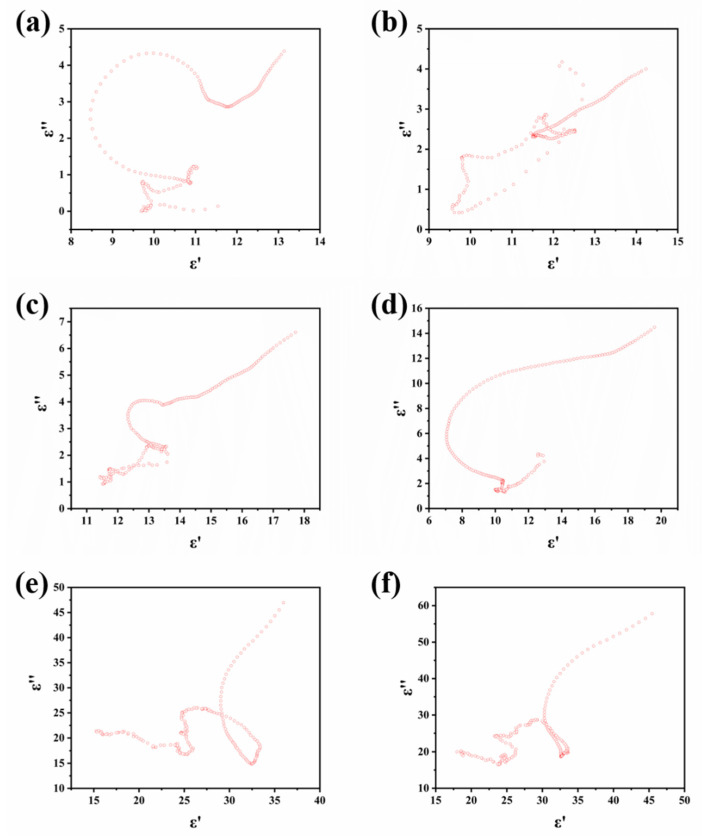
Cole–Cole semicircle of samples (**a**) CuPc-600, (**b**) CuPc-700, (**c**) CuPc-800, (**d**) CuPc-900, (**e**) CuPc-900/HCl, and (**f**) Pc.

The reflection loss (*R_L_*) is broadly used to evaluate the EMW absorption performance. According to transmission line theory, *R_L_* values were defined by the equation:

(1) $$Z_{\mathrm{in}}=Z_{0}\sqrt{\frac{\mu_{r}}{\varepsilon_{r}}}\tan h\left[j\frac{2\pi fd}{c}{\left(\mu_{r}\varepsilon_{r}\right)}^{1/2}\right]$$

(2) $$R_{L}=20\log_{10}\left|\frac{Z_{\mathrm{in}}-Z_{0}}{Z_{\mathrm{in}}+Z_{0}}\right|$$

where *Z*_in_ is the input impedance, *Z*_0_ is the air impedance, *f* is the frequency of the EMW, *d* is the thickness of the absorber, and *c* represents the velocity of light in vacuum. Figure 6 shows the two-dimensional and three-dimensional reflection loss plots of copper phthalocyanine carbon nanosheet samples at different cracking temperatures. The x-axis, y-axis, and z-axis of the four three-dimensional diagrams represent the frequency (GHz), thickness (mm), and reflection loss (dB), respectively. Generally speaking, when the reflection loss is less than −10 dB, the absorption of electromagnetic waves can reach more than 90%, at which time this material is sufficient to meet the requirements of practical applications, so the bandwidth where the reflection loss is less than −10 dB is defined as the effective absorption bandwidth. Both the maximum absorption bandwidth and the minimum reflection loss are important criteria to measure the absorption performance of a material. For the four samples, as the thickness of the coaxial ring increases, it can be seen that the frequency point where the value of the maximum reflection loss appears gradually moves to the lower frequency region. CuPc-600 showed a maximum absorption bandwidth of 2.56 GHz (between 6.88 and 9.44 GHz) at a thickness of 3 mm, and the minimum reflection loss can reach −38.4 dB at 7.68 GHz (Figure 6a,b). CuPc-700 has a maximum absorption bandwidth of 2.72 GHz (between 12.16 and 14.88 GHz) when the thickness is 1.7 mm, and the maximum reflection loss reaches −15.8 dB when the thickness is 5 mm (Figure 6c,d). As shown in Figure 6e,f, CuPc-800 has a maximum reflection loss of 3.2 GHz (12–15.2 GHz) at a thickness of 1.6 mm. When the thickness is 4 mm, the minimum reflection loss occurs and the R_Lmin_ reaches −37.4 dB. Figure 7a–d presents the *ε*′-*ε*″ curves of samples CuPc-600, CuPc-700, CuPc-800, and CuPc-900, respectively. Due to the different electronegativity between carbon atom and oxygen atom, the oxygen-containing chemical bonds on the surface of samples such as C=O could produce electronic dipole polarization. As the temperature increases, the number of oxygen-containing functional groups on the surface of the sample may gradually decrease. Previous Raman results showed that the percentage of defective carbon in the material keeps increasing as the temperature increases and the defective carbon also produces dipole polarization. Coupled with the fact that copper particles continue to aggregate with increasing temperature and form a Cu/CuO/carbon trilayer structure, these three factors lead to some differences in the electromagnetic wave loss mechanisms of the samples as the processing temperature changes. Compared to CuPc-600 and CuPc-700, the effective absorption bandwidth of CuPc-800 is significantly increased at a thinner thickness. This can be explained by the previous SEM image: the carbon nanosheet material was produced at a processing temperature of 800 °C, and the interlayer reflection between the layers of this sheet structure can effectively enhance the absorption of the incident electromagnetic waves by the material. As shown in Figure 6g,h, CuPc-900 has a maximum absorption bandwidth of 4.48 GHz (between 12.08 and 16.56 GHz) with a thickness of 1.7 mm and a minimum reflection loss of −25.3 dB when the thickness is 2 mm. It can be seen that the maximum absorption bandwidth of CuPc-900 has been significantly improved, which confirms the analysis of the previous SEM image. CuPc-900 has a unique wave texture structure, and has three interfaces of carbon nanosheets, copper oxides, and copper to reflect and transmit the incident electromagnetic waves. Compared with CuPc-600, its maximum absorption bandwidth is almost doubled.

**Figure 8:**
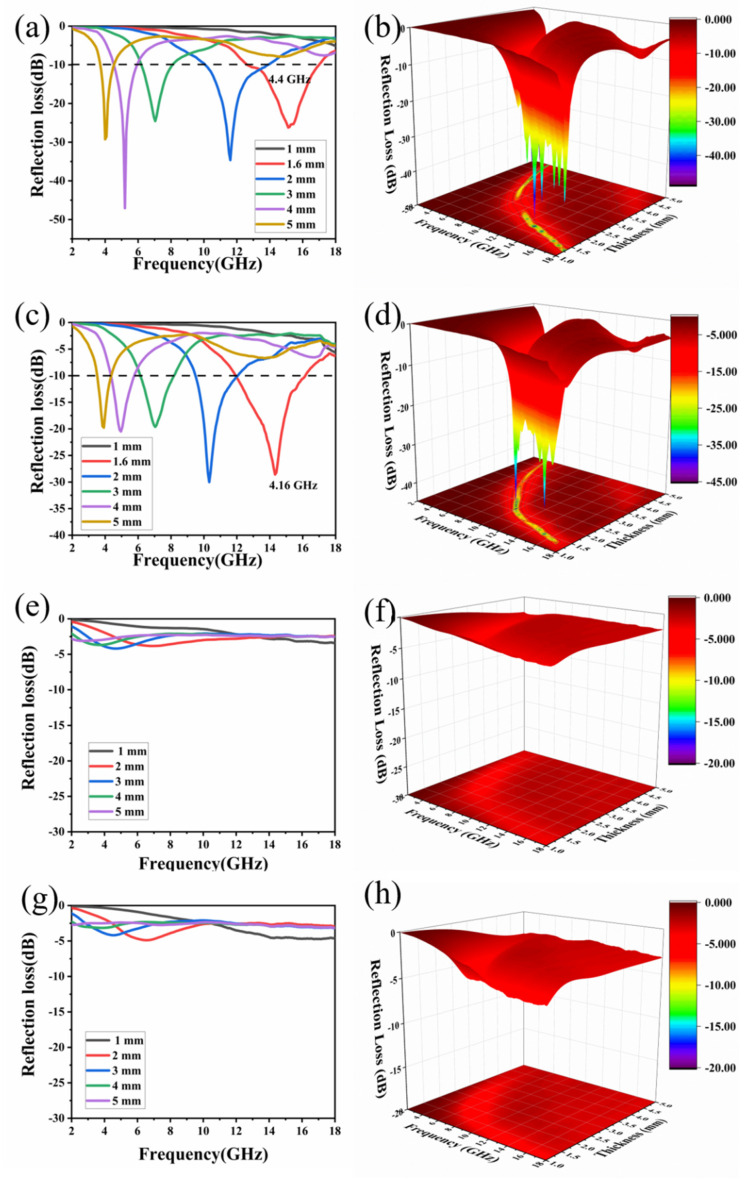
Reflection loss of (**a**,**b**) CuPc after 5% NaCl soaking, (**c**,**d**) CuPc after 10% NaCl soaking, (**e**,**f**) CuPc after 1 M HCl soaking, and (**g**,**h**) Pc.

Finally, it was also investigated whether the CuPc-900 maintained a stable performance after soaking in salt water and HCl. Figure 8a–d show the wave absorption performance of CuPc-900 after 3 weeks of immersion in 5% NaCl and 10% NaCl, respectively. After soaking in 5% NaCl, CuPc-900 has a maximum absorption bandwidth of 4.4 GHz (between 12.64 and 17.04 GHz) with a thickness of 1.6 mm. After soaking in 5% NaCl, CuPc-900 showed a maximum absorption bandwidth of 4.16 GHz (between 12 and 16.16 GHz) at a thickness of 1.6 mm. This is not much different from the previous data, which shows that CuPc-900 can maintain a stable wave absorption performance in a heavy salt environment, which provides a new direction for the selection of absorbing materials in some extreme environments. In order to demonstrate the contribution of the multilayer interfacial reflection of Cu/CuO/carbon nanosheet composites to the wave absorption performance, CuPc-900 was soaked in 1 M HCl and then tested for the wave absorption performance. Plus, the wave absorption performance of phthalocyanine was tested in the case of sintering at 900 °C to further analyze the wave absorption mechanism of CuPc-900. Figure 7e and Figure 8e,f depict the CuPc-900 Cole–Cole curves and the two-dimensional and three-dimensional absorption performance plots after washing with 1 M HCl, respectively. Figure 7f and Figure 8g,h depict the phthalocyanine Cole–Cole curves and the two-dimensional and three-dimensional wave absorption performance plots after treatment with 900 °C, respectively. The general pattern of the Cole–Cole curve is the same for both, indicating that the loss mechanism of CuPc-900 after washing off copper is not much different from that of pure carbon nanosheets. What is more, we can clearly find a long tail in the curves, which manifests the contribution of the conduction loss. Compared with CuPc-900, it appears to be very different, which shows that the composite structure of Cu/CuO/carbon can really change the loss mechanism of the whole material. The absorbing performance of CuPc-900/HCl and Pc is very poor, not reaching -10 dB absorption in the full waveband, but with the composite structure of Cu/CuO/carbon, the Debye relaxation loss is enhanced, and the real and imaginary parts of the dielectric constant are reduced, which improves the impedance matching and greatly improves the absorbing performance of the material.

## 3. Materials and Methods

### 3.1. Materials

Copper phthalocyanine (CuPc) was purchased from Nantong Zhengyan New Material Technology Co., Ltd., Nantong, China. Paraffin was purchased from Shanghai Hualing rehabilitation Machinery Factory., Shanghai, China. NaCl and HCl were purchased from Chengdu Kolon Chemical Co. Ltd., Chengdu, China. All the chemicals were used as received unless otherwise stated. phthalocyanine (Pc) was purchased from Shanghai Aladdin Biochemical Technology Co., Ltd., Shanghai, China.

### 3.2. Preparation of Cu/CuO/Carbon

Copper phthalocyanine (CuPc) (5.0 g) was pyrolyzed into a crucible, and then it was wrapped with copper foil and placed in a tube furnace. The specific warming procedure is as follows: first, ventilated with Ar at 30 °C for 1 h; heated up to 300 °C at a rate of 5 °C per min and kept at 300 °C for 1 h; heated up to 900 °C at a rate of 2 °C per min and kept at 900 °C for 8 h; finally, naturally cooled to room temperature. Then, the final maximum temperature of the above experimental conditions was changed by different cracking temperatures for the comparison tests, and 600 °C, 700 °C, and 800 °C were used, respectively. Depending on the final pyrolysis temperature, the samples were named CuPc-600, CuPc-700, CuPc-800, and CuPc-900.

To test the corrosion resistance of the material, CuPc-900 was immersed in 5 wt.% NaCl solution, 10 wt.% NaCl solution, and 1 M HCl for three weeks and then washed with deionized water and dried. The samples were named CuPc-900/5NaCl, CuPc-900/10NaCl, and CuPc-900/HCl, respectively. Finally, phthalocyanine was sintered at 900 °C using the same sintering method as a comparison sample, and named as Pc.

### 3.3. Material Characterization

The morphologies of the samples were imaged using a JSM 6490LV from JEOL (BEIJING) Co., Ltd., Beijing, China scanning electron microscope (SEM). High-resolution transmission electron microscopy (HRTEM) images were collected on a Technai G2 20. X-ray photoelectron spectroscopy (XPS) was performed using an X-ray photoelectron spectrometer (XPS, Thermo Scientific K-Alpha+) from ThermoFisher Technology (China) Co., Ltd., shanghai, China. X-ray diffraction (XRD) was conducted with a X’ Pert PRO diffractometer using Cu Kα radiation (λ = 1.54056 Å).

### 3.4. Microwave Absorption Measurement

A vector network analyzer (E5063A) from Agilent Technology (China) Co., Ltd., Beijing, China with a coaxial method in the 2.0–18 GHz range was used to measure the EMWs absorption characteristics of the obtained materials. The coaxial line method is used to measure the absorbing properties of the material. The material powder sample and the solid paraffin block were weighed according to a certain mass percentage, then heated to 70 °C, and mixed well. Finally, used coaxial ring preparation mold to sharp mixture as a cylindrical ring with an inner diameter of 3.04 mm and an outer diameter of 7.0 mm.

## 4. Conclusions

A simple high-temperature solid-phase cracking method was used to prepare composites of copper particles and nitrogen-crosslinked carbon nanosheets whose multilayer interfaces reflect and transmit electromagnetic waves, leading to excellent electromagnetic absorption properties. The prepared material has a maximum absorption bandwidth of 4.48 GHz at an exceedingly thin thickness of 1.7 mm and a minimum reflection loss of -25.3 dB when the thickness is 2 mm when the cracking temperature is 900 °C. In general, the composite with simple preparation and special structure can become an ideal candidate material with good electromagnetic wave absorption properties.

## Data Availability

The data presented in this study are available on request from the corresponding author.

## Sample Availability

Samples of the compounds are available from the authors.

## References

[1] Gao X., Xue W., Ye W., Zhao R., Ma X., He D. (2021). Large-scale synthesis of nitrogen doped graphene/carbon nanotube composites by solid-state pyrolysis of nickel phthalocyanine and its synergistic effect for microwave absorption properties. Mater. Lett..

[2] He D., Xue W., Li Z., Marsden A.J., Prestat E., Hu W., Zhao R., Bissett M.A. (2018). Long-range oriented graphene-like nanosheets with corrugated structure. Chem. Commun..

[3] Li Z., Mi H., Bai Z., Ji C., Sun L., Gao S., Qiu J. (2019). Sustainable biowaste strategy to fabricate dual-doped carbon frameworks with remarkable performance for flexible solid-state supercapacitors. J. Power Sources.

[4] Ruan Z., Qin J., Li Z. (2015). The partially controllable growth trend of carbon nanoparticles in solid-state pyrolysis of organometallic precursor by introducing POSS units, and their magnetic properties. RSC Adv..

[5] Ruan Z., Rong W., Li Q., Li Z. (2015). Oxygen as the growth enhancer of carbon nanotubes in solid-state pyrolysis of organometallic precursors. Carbon.

[6] Cao W., Ma C., Tan S., Ma M., Wan P., Chen F. (2019). Ultrathin and Flexible CNTs/MXene/Cellulose Nanofibrils Composite Paper for Electromagnetic Interference Shielding. Nanomicro Lett..

[7] Wang L., Huang Y., Li C., Chen J., Sun X. (2015). A facile one-pot method to synthesize a three-dimensional graphene@carbon nanotube composite as a high-efficiency microwave absorber. Phys. Chem. Chem. Phys..

[8] Wang S., Xu Y., Fu R., Zhu H., Jiao Q., Feng T., Feng C., Shi D., Li H., Zhao Y. (2019). Rational Construction of Hierarchically Porous Fe-Co/N-Doped Carbon/rGO Composites for Broadband Microwave Absorption. Nanomicro Lett..

[9] Zhao H., Cheng Y., Liu W., Yang L., Zhang B., Wang L.P., Ji G., Xu Z.J. (2019). Biomass-Derived Porous Carbon-Based Nanostructures for Microwave Absorption. Nanomicro Lett..

[10] Feng W., Wang Y., Chen J., Wang L., Guo L., Ouyang J., Jia D., Zhou Y. (2016). Reduced graphene oxide decorated with in-situ growing ZnO nanocrystals: Facile synthesis and enhanced microwave absorption properties. Carbon.

[11] Kong L., Yin X., Xu H., Yuan X., Wang T., Xu Z., Huang J., Yang R., Fan H. (2019). Powerful absorbing and lightweight electromagnetic shielding CNTs/RGO composite. Carbon.

[12] Shu R., Zhang J., Guo C., Wu Y., Wan Z., Shi J., Liu Y., Zheng M. (2020). Facile synthesis of nitrogen-doped reduced graphene oxide/nickel-zinc ferrite composites as high-performance microwave absorbers in the X-band. Chem. Eng. J..

[13] Wang H., Meng F., Huang F., Jing C., Li Y., Wei W., Zhou Z. (2019). Interface Modulating CNTs@PANi Hybrids by Controlled Unzipping of the Walls of CNTs To Achieve Tunable High-Performance Microwave Absorption. ACS Appl. Mater. Interfaces.

[14] Wang X., Lu Y., Zhu T., Chang S., Wang W. (2020). CoFe_2_O_4_/N-doped reduced graphene oxide aerogels for high-performance microwave absorption. Chem. Eng. J..

[15] Yin Y., Liu X., Wei X., Yu R., Shui J. (2016). Porous CNTs/Co Composite Derived from Zeolitic Imidazolate Framework: A Lightweight, Ultrathin, and Highly Efficient Electromagnetic Wave Absorber. ACS Appl. Mater. Interfaces.

[16] Yuan Y., Wei S., Liang Y., Wang B., Wang Y., Xin W., Wang X., Zhang Y. (2021). Solvothermal assisted synthesis of CoFe2O4/CNTs nanocomposite and their enhanced microwave absorbing properties. J. Alloys Compd..

[17] Zhang H., Pang H., Duan Y., Zhang W., Wang T., Zhang X. (2021). Facile morphology controllable synthesis of zinc oxide decorated carbon nanotubes with enhanced microwave absorption. J. Mater. Sci.: Mater. Electron..

[18] Chaiyo S., Mehmeti E., Siangproh W., Hoang T.L., Nguyen H.P., Chailapakul O., Kalcher K. (2018). Non-enzymatic electrochemical detection of glucose with a disposable paper-based sensor using a cobalt phthalocyanine-ionic liquid-graphene composite. Biosens. Bioelectron..

[19] Duan F., Hu M., Guo C., Song Y., Wang M., He L., Zhang Z., Pettinari R., Zhou L. (2020). Chromium-based metal-organic framework embedded with cobalt phthalocyanine for the sensitively impedimetric cytosensing of colorectal cancer (CT26) cells and cell imaging. Chem. Eng. J..

[20] Mahato A.K., Bharti D., Varun I., Saxena P., Raghuwanshi V., Tiwari S.P. (2021). UV assisted non-volatile memory behaviour using Copper (II) phthalocyanine based organic field-effect transistors. Org. Electron..

[21] Marchis T., Avetta P., Bianco-Prevot A., Fabbri D., Viscardi G., Laurenti E. (2011). Oxidative degradation of Remazol Turquoise Blue G 133 by soybean peroxidase. J. Inorg. Biochem..

[22] Ruiz G.T., Ferraudi G., Lappin A.G. (2009). Excited state vs. photoinduced charge separation in bundles of a polyamide containing pendant AlIII phthalocyaninetetrasulfonate groups. Potential applications to photocatalysis. J. Photochem. Photobiol. A Chem..

[23] Sevim A.M., Ilgün C., Gül A. (2011). Preparation of heterogeneous phthalocyanine catalysts by cotton fabric dyeing. Dyes Pigments.

[24] Shukla P., Yadav S., Patel M.S., Kumar P., Kumar N., Kumar L. (2021). The effects of cesium lead bromide quantum dots on the performance of copper phthalocyanine-based organic field-effect transistors. Nanotechnology.

[25] Zhang M., Shao C., Guo Z., Zhang Z., Mu J., Zhang P., Cao T., Liu Y. (2011). Highly efficient decomposition of organic dye by aqueous-solid phase transfer and in situ photocatalysis using hierarchical copper phthalocyanine hollow spheres. ACS Appl. Mater. Interfaces.

[26] Xue W.-D., Zhao R. (2014). A simple approach towards nitrogen-doped graphene and metal/graphene by solid-state pyrolysis of metal phthalocyanine. New J. Chem..

